# Parrot Beak Nail: Case Report and Review of Parrot Beak Nail Dystrophy

**DOI:** 10.7759/cureus.15974

**Published:** 2021-06-27

**Authors:** Parnia Forouzan, Philip R Cohen

**Affiliations:** 1 Medicine, McGovern Medical School, University of Texas Health Science Center at Houston, Houston, USA; 2 Dermatology, San Diego Family Dermatology, National City, USA

**Keywords:** beak, deformity, digit, dystrophy, finger, nail, onychodystrophy, parrot, toe, treatment

## Abstract

Parrot beak nail dystrophy is an excessive forward curvature of the nail plate that can affect both fingernails and toenails. Few cases have been reported since its original description in 1971; however, the incidence is estimated to be 2.5% in healthy individuals. Although the pathogenesis has not yet been established, parrot beak nail has been associated with chronic crack cocaine use, congenital bone or soft tissue abnormalities, other nail dystrophies, peripheral neuropathy, systemic sclerosis, and trauma to the nail. We describe an 86-year-old man with dementia and neuropathy who presented with an unperceived parrot beak nail of his left fourth toenail and concurrent onycholysis of his left great toenail. He had stopped visits with his podiatrist for nail care, which fostered the growth of these nail dystrophies. Our patient’s parrot beak nail was successfully treated with nail clipping and regular nail maintenance to prevent its recurrence. The associated conditions, etiologies, and treatment of parrot beak nails are discussed.

## Introduction

Nail dystrophies can be congenital or acquired. Hence, some conditions frequently present in pediatric patients, whereas others are more commonly observed in elderly individuals. The condition can be isolated to only the nails or a manifestation of a systemic disorder or drug reaction [1].

Parrot beak nail is a forward over-curvature of the nail plate. It can affect the fingernails or toenails and can involve a single or multiple digits. Other nail dystrophies, such as onycholysis and subungual hematomas, can be simultaneously present as well [2].

An 86-year-old man presented with dystrophy of his toenails on his left foot. The fourth toe demonstrated over-curvature of the nail (referred to as parrot beak nail) with concurrent thickening and distal shedding of his great toenail. The associations, features, and treatment of parrot beak nail dystrophy are reviewed.

## Case presentation

An 86-year-old man who is regularly followed with total body skin checks presented for his periodic visit. His medical history is significant for chronic kidney disease, chronic obstructive pulmonary disease (COPD), dementia, hypothyroidism, and prostate cancer. His medications include aspirin, donepezil, leuprolide acetate, and tamsulosin.

He also has a history of actinic keratoses, basal cell carcinoma, and squamous cell carcinoma. The actinic keratoses were treated with cryotherapy; the skin cancers were treated with Mohs micrographic surgery. Cutaneous examination revealed no new or recurrent actinic keratoses or skin neoplasms.

**Figure 1:**
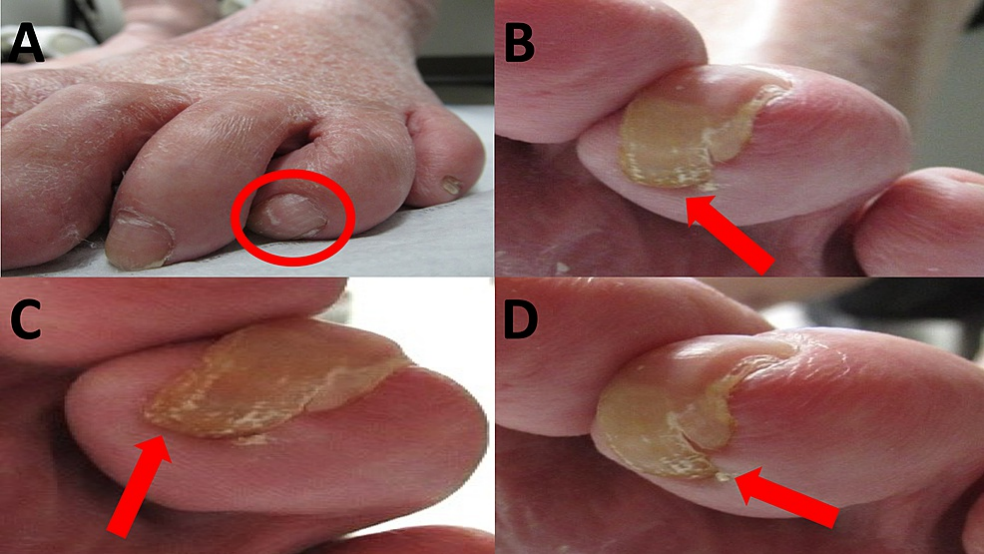
Parrot beak nail dystrophy of the left fourth toenail An 86-year-old man presented with a parrot beak nail of the left fourth toe. The nail dystrophy (red circle) was not evident when viewed from the front (A); however, over-curvature was apparent (red arrows) when viewed from the lateral (B) and inferior surfaces (C) of the left foot. Impingement of the nail (D) into the soft pulp of the digit (red arrow) was also observed without surrounding infection or pain.

Examination of his finger and toenails was performed. His left foot showed forward over-curvature of his fourth toenail with an extension of the nail into the distal soft pulp of the digit (Figure 1). This is commonly referred to as a parrot beak nail.

**Figure 2:**
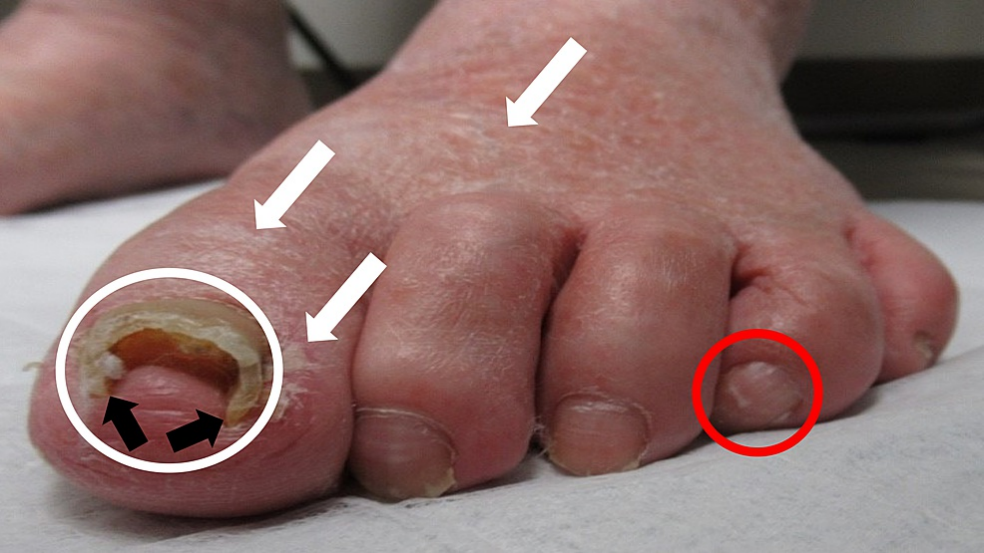
Onycholysis of the left great toenail and xerosis of the skin concurrent with parrot beak nail of the left fourth toenail Thickening of the left great toenail with distal onycholysis (white circle) was observed on the same foot as the parrot beak nail (red circle). A pincer nail deformity, demonstrated by pinching of the distal nail bed secondary to excessive transverse nail curvature (black arrows), of the great toenail was noted. In addition, xerosis (white arrows) of the skin on the dorsal foot extending onto the toe and lateral proximal nail fold was present.

In addition, there was distal onycholysis and pincer nail dystrophy of the left great toenail (Figure 2). Xerosis of the dorsal foot and toes was also noted. The toenails on his right foot and all fingernails were normal. 

The patient was unaware of his toenail elongation, potentially due to androgen deprivation therapy-induced peripheral neuropathy. He is unable to cut his own toenails and periodically sees a podiatrist to maintain them. His last nail cut was more than six months ago in early 2020 with no scheduled follow-up due to the coronavirus pandemic.

**Figure 3:**
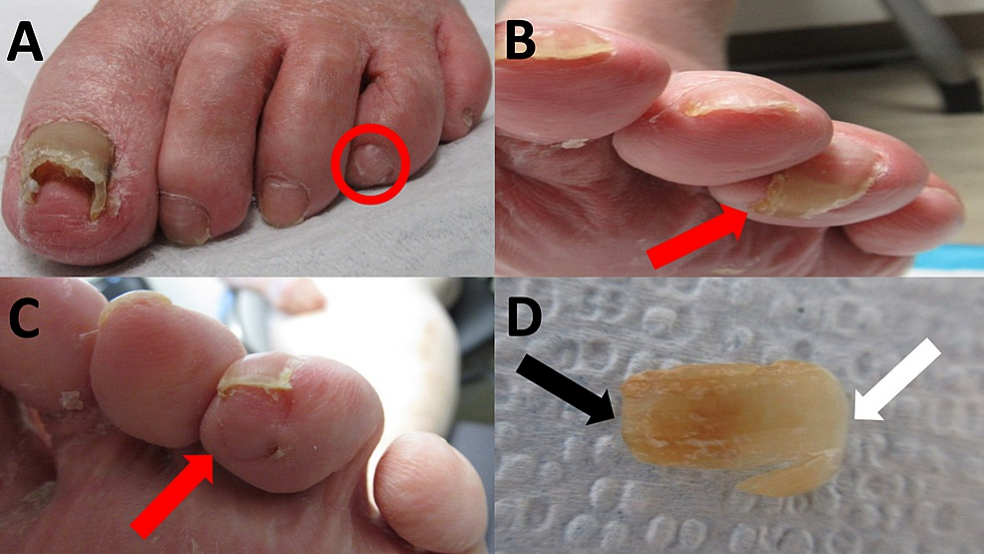
Pre-treatment and post-therapy (clipping) of the left fourth toenail of an 86-year-old man with a parrot beak nail deformity An anterior (A) and inferior (B) view of the parrot beak nail dystrophy (red circle, red arrow) before nail clipping. After treatment (C), there was a residual impression (red arrow) on the soft pulp of the digit without underlying infection. The removed parrot beak nail (D) shows over-curvature from the proximal (white arrow) to the distal (black arrow) nail plate.

Management of the parrot beak nail consisted of distal nail plate removal using nail clippers. The lingering impression of the removed portion of the nail plate was evident in the distal soft pulp (Figure 3); however, there was no underlying infection. To prevent its recurrence, the patient was advised to contact his podiatrist for continued toenail maintenance.

## Discussion

The parrot beak nail is an uncommonly reported nail dystrophy characterized by excessive curvature of the nail plate. It was first observed and reported by Kandil in 1971. Since then, there have been six additional manuscripts published including this case report (Table 1) [2-7].

**Table 1:** Reports of parrot beak nail dystrophy Abbreviations: CR, current report; pbn, parrot beak nail

Author, year published	Comments	References
Kandil, 1971	Kandil coined the term pbn when a 38-year-old housewife presented with symmetric, bilateral over-curvature of her third and fourth fingernails after she stopped washing her utensils. The patient was successfully treated with water submersion of her affected nails.	[3]
Kurokawa et al., 1993	An 11-year-old girl with pbn of the third and fourth toenails and congenital absence of distal soft tissue of two toes is described. Also, a 17-year-old girl with pbn of the fourth toe and bony hypoplasia of one toe is reported. Both patients were successfully treated with the recession of the nail, nailbed, and nail matrix.	[4]
Payne-James et al., 2007	Eight women (24 to 40-years-old) who were chronic crack cocaine users were found to have a “pseudosclerodermatous” triad of distal pulp atrophy, pbn, and perniosis due to proposed cocaine-induced vasoconstriction of the digits.	[5]
Desai et al., 2011	A 55-year-old woman with no significant past medical history had pbn of all of her fingernails. Treatment with water submersion provided temporary correction. Her pbn recurred when she allowed her fingernails to grow.	[6]
Marie et al., 2017	129 patients with scleroderma and 80 healthy individuals were observed for fingernail abnormalities. Of these, 40 patients with scleroderma and two healthy subjects had pbn of their fingernails.	[7]
Chen and Cohen, 2017	Ten men with pbn of their toenails were reported among 436 patients seen at a dermatology clinic. Concurrent onycholysis and subungual hematomas were noted in some patients. They were successfully treated through clipping of their affected toenails with regular maintenance.	[2]
Forouzan and Cohen, 2021	An 86-year-old man with a history of dementia and prostate cancer had pbn of his fourth toenail with concurrent onycholysis of his great toenail. He was successfully treated with clipping of the affected nail and regular nail maintenance.	[CR]

Sixty-five patients with parrot beak nails have been reported, including our own. Ages at presentation ranged from 11 to 89-years-old. The mean age of presentation was 32-years-old in women and 76-years-old in men [2-7].

Parrot beak fingernails have been observed in 52 individuals. In contrast, parrot beak toenails have been reported in 13 individuals. To date, an individual with both finger and toe parrot beak nails has not been described [2-7].

Twelve women and 11 men have been documented in case reports. Forty-two patients did not have their genders specified. Notably, 10 of the 12 women described had parrot beak nails of their fingers, whereas all of the men had parrot beak nails of their toes [2-7].

Chen and Cohen found 2.1% of 436 patients presenting to a dermatology clinic had parrot beak nail dystrophy. Marie et al. recorded an incidence of 2.5% among 80 healthy subjects. In individuals with systemic sclerosis, the incidence of parrot beak nails was 31% [2,7].

Parrot beak nail presents as excessive forward curvature of the distal nail plate. Depending on the duration of development, it continues to extend over the distal tip of the digit and through the plantar surface of the finger or toe pad. Unlike other nail dystrophies, such as clubbing or pachyonychia, the proximal nail plate, nail bed, and soft tissue, are usually normal [2].

Patients can be unaware of their nail dystrophy, as it is typically asymptomatic. However, if allowed to grow, the parrot beak nail can cause impingement of the soft tissue pulp with potential for skin breakdown, infection, or both. This can lead to functional impairment or pain [2].

Parrot beak nails of the fingers have been more commonly observed. Notably, fingernails affected by parrot beak nails usually occur in individuals without nail dystrophy-associated conditions and is therefore referred to as idiopathic (Table 2) [3,5-7]. It is less commonly associated with other conditions such as chronic cocaine use and systemic sclerosis [2-7].

**Table 2:** Conditions associated with parrot beak fingernails

Associated conditions	References
Absence of associated condition (idiopathic)	[3,6]
Chronic crack cocaine use	[5]
Systemic sclerosis	[7]

In contrast, patients with parrot beak nails of the toes had more associated conditions such as congenital abnormalities, nail dystrophies, neuropathies, and systemic conditions (Table 3) [2,4]. Some conditions, such as cancer and coronary artery disease, may not have a pathogenic effect on the development of parrot beak nails; however, others such as peripheral neuropathy can influence the growth of parrot beak nails through the absence of detection [2-7].

**Table 3:** Conditions associated with parrot beak toenails Abbreviations: CR, current report ^a^Both bladder, prostate, and skin cancer were observed in patients with parrot beak nail deformity [2,CR]. ^b^Hammer toe and an overlying fifth toe were reported [2]. ^c^Concurrent nail dystrophies include longitudinal erythronychia, onychauxis, onychogryphosis, onycholysis, pincer nail, and subungual hematoma [2,CR]. ^d^Neuropathy occurred secondary to androgen deprivation therapy, diabetes, and spinal stenosis [2,CR].

Associated conditions	References
Absence of associated condition (idiopathic)	[2]
Bony hypoplasia	[4]
Cancer^a^	[2,CR]
Chronic kidney disease	[CR]
Chronic obstructive pulmonary disease	[CR]
Coronary artery disease	[2]
Dementia	[CR]
Digit deformity^b^	[2]
Hypertension	[CR]
Hypothyroidism	[CR]
Lymphoplasmacytic sclerosing pancreatitis	[2]
Multiple system atrophy	[2]
Nail dystrophy^c^	[2,CR]
Neuropathy^d^	[2,CR]
Soft tissue hypoplasia	[4]

A parrot beak nail can concurrently occur with other nail dystrophies. Onychauxis, onychogryphosis, onycholysis, and subungual hematomas were noted in a study of 10 patients [2]. Our patient had accompanying thickening of his great toenail with distal onycholysis.

The etiology of parrot beak nails is still uncertain (Table 4) [2,4-5,7-9]. Presumed congenital parrot beak nail dystrophy has been observed in an 11-year-old girl and a 17-year-old girl with concurrent digit abnormalities. However, most cases of parrot beak nails are assumed to be secondary to chronic cocaine use, digit injury, or systemic sclerosis [2,4-5,7-9].

**Table 4:** Proposed etiologies for parrot beak nail dystrophy Abbreviations: pbn, parrot beak nail

Potential etiology	Comments	References
Chronic crack cocaine use	Nearly continuous and chronic crack cocaine use was associated with the development of pbn in eight women. Pbn was hypothesized to be related to prolonged cocaine-induced vasoconstriction and peripheral ischemia of the distal digit. Chronic cocaine use has been associated with a triad of distal pulp atrophy, pbn, and perniosis resembling scleroderma. Amphetamine use has also been implicated as an etiology of parrot beak nails through a similar mechanism.	[2,5]
Congenital bone or soft tissue abnormalities	Presumed congenital pbn was observed in two girls, an 11-year-old and a 17-year-old, with concurrent congenital soft tissue and bony hypoplasia of adjacent digits.	[4]
Systemic sclerosis	Vasoconstriction and subsequent digital ischemia, similar to chronic cocaine use, is the proposed mechanism. The incidence of pbn in individuals with systemic sclerosis is 15 times greater than in healthy individuals.	[7]
Trauma to the digit or nail	Trauma includes digit amputation, repetitive distal digit injury, and tight surgical closure of the fingertip due to loss of nail support. Trauma-associated pbn can be circumvented through relocation of the nail complex or the creation of a tension-free surgical closure of the fingertip with a hypodermic needle.	[2,8,9]

The pathogenesis of parrot beak nails is also unknown. However, it is postulated to arise from abnormal phospholipid distribution in the nail plate. The nail plate consists of three zones that differ in their relative composition of calcium, phospholipid, and sulfhydryl groups [2,10].

In particular, hydrophobic interactions between these zones have been hypothesized as the cause of parrot beak nails. The nail over-curvature temporarily corrects after the submergence of the affected nail in water for up to 30 minutes. Prolonged exposure to water may overcome the abnormal hydrophobic nail plate zone interactions, normalizing the curvature of the nail [2].

Chronic vasoconstrictive ischemia is another hypothesized pathogenesis for parrot beak nails. This was proposed in a study of eight women with a long history of cocaine use who had similar digital abnormalities including parrot beak nails. In addition, there is a greater incidence of parrot beak nails in individuals with systemic sclerosis, which is also associated with prolonged vasoconstriction and subsequent digit ischemia [2,5].

Patients at risk for parrot beak nails include older individuals and those with sensory impairment of their distal digits (Table 5) [2,8-9,11]. Peripheral neuropathy can be associated with autoimmune conditions, cancer, endocrine dysfunction, medications, and nutritional deficiencies. These can promote the development of parrot beak nails because individuals such as our patient with androgen deprivation therapy-induced neuropathy may unknowingly neglect or injure their digits [2,11].

**Table 5:** Risk factors for parrot beak nail dystrophy Abbreviations: HIV, human immunodeficiency virus; pbn, parrot beak nail ^a^Neuropathy can occur secondary to autoimmune conditions (celiac disease, connective tissue disease, sarcoidosis, and vasculitis), cancer, endocrine dysfunction (chronic kidney disease, diabetes mellitus, hyperthyroidism, hypothyroidism, and liver disease), infections (HIV and leprosy), medications (amiodarone, chemotherapy, colchicine, isoniazid, leflunomide, and phenytoin), nutritional deficiencies (copper and vitamins B1, B6, B12, and E). Parkinson’s disease or parkinsonian features could also enhance the development of parrot beak nails due to increased risk of trauma and neuropathy [2,11].

Risk factor	Comments	References
Musculoskeletal limitations	Conditions that can physically limit maintenance of nail care, fostering the growth of pbn.	[2]
Neurodegeneration	Pathologic or physiologic age-related neurodegeneration increases the risk of falls which can promote the development of injury-induced pbn.	[2,8,9]
Neuropathy	Primary or secondary neuropathy^a^ can cause neglect or increased trauma of distal digits, increasing the risk for pbn.	[2,11]

Individuals prone to digit trauma and falls are also at greater risk for injury-induced parrot beak nails. This includes our elderly patient with dementia and individuals with neurodegeneration and neuropathy. In addition, orthopedic limitations can promote injury or neglect of the nail, enhancing the risk of developing parrot beak nails [2,8-9].

A parrot beak nail can be treated by soaking the affected nail in water to temporarily normalize its curvature before clipping the abnormal nail. A free nail edge can then be filed to create a smooth edge. Periodic nail cutting and regular nail care should also be recommended to prevent repeated parrot beak nail growth and its potential consequences [6].

Although not typically advocated, parrot beak nails can be cured through the removal of the nail plate and ablation of the nail matrix, which prevents new nail growth and eliminates the potential for recurrence. A surgical technique involving proximal relocation of the nailbed, nail matrix, and nail plate has also been used to treat congenital parrot beak nails. These techniques may be beneficial in individuals with recurrent and symptomatic parrot beak nails [2,4].

## Conclusions

Nail dystrophies can be acquired or congenital and involve the fingers and/or toes. A parrot beak nail presents as a forward over-curvature of the nail plate with an incidence of 2.5% in healthy individuals. Although first described in 1971, parrot beak nails have rarely been described in the literature. We suspect the incidence to be higher among healthy individuals due to underreporting. A parrot beak nail is usually asymptomatic but can lead to functional impairment, infection, pain, or skin breakdown of the distal digit if not prevented or treated. The parrot beak nail has been associated with other nail deformities and systemic sclerosis. The pathogenesis is uncertain but potential etiologies include congenital abnormalities, chronic cocaine use, systemic sclerosis, and trauma of the distal digit. Individuals who have these conditions, neurodegeneration, or peripheral neuropathy are at greater risk of developing parrot beak nails. It is most commonly treated with water submersion and clipping of the affected nail with regular nail maintenance to prevent a recurrence.
